# Psychometric properties of the Dutch version of the Evidence-Based Practice Attitude Scale (EBPAS)

**DOI:** 10.1186/s12961-015-0058-z

**Published:** 2015-11-16

**Authors:** Maartje A. M. S. van Sonsbeek, Giel J. M. Hutschemaekers, Jan W. Veerman, Marloes Kleinjan, Gregory A. Aarons, Bea G. Tiemens

**Affiliations:** Behavioural Science Institute, Radboud University, Montessorilaan 3, 6525 HR Nijmegen, The Netherlands; Pro Persona Centre for Education & Science (ProCES), Postbus 27, 6870 AA Renkum, The Netherlands; Department of Epidemiology, Trimbos Institute, Da Costakade 45, 3521 VS Utrecht, The Netherlands; Department of Psychiatry, University of California, 9500 Gilman Drive, San Diego, La Jolla, CA 92093-0812 USA; Child and Adolescent Services Research Center, 3020 Children’s Way, San Diego, CA 92123 USA

**Keywords:** Attitude, EBPAS, Evidence-based practice, Professionals, Youth care

## Abstract

**Background:**

The Evidence-Based Practice Attitude Scale (EBPAS) was developed in the United States to assess attitudes of mental health and welfare professionals toward evidence-based interventions. Although the EBPAS has been translated in different languages and is being used in several countries, all research on the psychometric properties of the EBPAS within youth care has been carried out in the United States. The purpose of this study was to investigate the psychometric properties of the Dutch version of the EBPAS.

**Methods:**

After translation into Dutch, the Dutch version of the EBPAS was examined in a diverse sample of 270 youth care professionals working in five institutions in the Netherlands. We examined the factor structure with both exploratory and confirmatory factor analyses and the internal consistency reliability. We also conducted multiple linear regression analyses to examine the association of EBPAS scores with professionals’ characteristics. It was hypothesized that responses to the EBPAS items could be explained by one general factor plus four specific factors, good to excellent internal consistency reliability would be found, and EBPAS scores would vary by age, sex, and educational level.

**Results:**

The exploratory factor analysis suggested a four-factor solution according to the hypothesized dimensions: Requirements, Appeal, Openness, and Divergence. Cronbach’s alphas ranged from 0.67 to 0.89, and the overall scale alpha was 0.72. The confirmatory factor analyses confirmed the factor structure and suggested that the lower order EBPAS factors are indicators of a higher order construct. However, Divergence was not significantly correlated with any of the subscales or the total score. The confirmatory bifactor analysis endorsed that variance was explained both by a general attitude towards evidence-based interventions and by four specific factors. The regression analyses showed an association between EBPAS scores and youth care professionals’ age, sex, and educational level.

**Conclusions:**

The present study provides strong support for a structure with a general factor plus four specific factors and internal consistency reliability of the Dutch version of the EBPAS in a diverse sample of youth care professionals. Hence, the factor structure and reliability of the original version of the EBPAS seem generalizable to the Dutch version of the EBPAS.

## Background

The dissemination and implementation of evidence-based practice (EBP) to improve the quality of care and outcomes for clients and their families is a critical concern worldwide [1]. EBP is the integration of the best available research with clinical expertise in the context of client characteristics, culture, and preferences [2,3]. The purpose of EBP is to promote effective psychological practice and enhance public health by applying empirically supported principles of psychological assessment, case formulation, therapeutic relationship, and intervention. EBP also holds the promise to increase cost-effectiveness [4]. EBP may not only comprise the use of efficacious interventions, but may also include innovations such as data monitoring systems, alerts to target prescribing practices, and routine outcome monitoring (ROM) with feedback to clinicians [5-7].

Multiple factors at community, organizational, and individual levels influence the dissemination and implementation of EBP in real-world mental healthcare settings [8-10]. There is increasing evidence that the values and beliefs of professionals play an important role in the degree to which innovations are initiated and incorporated into common practice [11,12]. On the one hand, attitudes of professionals toward EBP can be a precursor to the decision of whether or not to try a new practice [13,14], and if professionals do decide to try a new practice, the affective or emotional component of attitudes can impact decision processes regarding the actual implementation and use of the innovation [12]. On the other hand, behaviour can influence attitudes [15]. Engaging in a behaviour, such as continued use of an evidence-based intervention until familiarity is developed, using a data monitoring system to track specific indicators of change, or attending collaborative meetings with one’s peers, may change attitudes and beliefs about EBP [5].

In order to tailor implementation efforts to meet the needs and characteristics of professionals in youth care institutions, we have to consider professionals’ attitudes toward adopting EBP [16,17]. Aarons [18] developed the Evidence-Based Practice Attitude Scale (EBPAS) to assess professionals’ attitudes toward adopting EBP in mental health and welfare settings. The EBPAS asks for professionals’ feelings about using evidence-based interventions, which are defined as new types of therapy or treatments and interventions with specific guidelines or components that are outlined in a manual or that are to be followed in a predetermined way. There are two other instruments to assess the attitudes of professionals toward adopting EBP. However, one is a non-validated survey questionnaire that was not designed to assess change over time [19]. The other was developed to measure professionals’ views about and implementation of the five steps of the EBP process [20] as defined by Sackett et al. [3]. Because most of our professionals are not trained in these five steps, we chose to use an instrument that assesses a general attitude toward using evidence-based interventions.

The EBPAS consists of 15 items measured on a 5-point Likert scale ranging from 0 (Not at all) to 4 (To a very great extent). The items of the EBPAS are organized into four dimensions. The Appeal subscale (four items) assesses the extent to which the professional would adopt an evidence-based intervention if it was intuitively appealing, could be used correctly, or was being used by colleagues who were happy with it. The Requirements subscale (three items) assesses the extent to which the professional would adopt an evidence-based intervention if it was required by the supervisor, agency, or state. The Openness subscale (four items) assesses the extent to which the professional is generally open to trying new interventions and would be willing to try or use more structured or manualized interventions. The Divergence subscale (four items) assesses the extent to which the professional perceives evidence-based interventions as not clinically useful and less important than clinical experience. The EBPAS total score is computed by first reverse scoring the Divergence scale item scores and then computing the overall mean [17]. The EBPAS total score represents one’s global attitude toward adoption of evidence-based interventions. The higher the score, the more positive the attitude toward evidence-based interventions.

Previous studies [16-18] confirmed the four-factor structure of the EBPAS in samples from the United States. These studies also suggested adequate internal consistency reliability for the EBPAS total score (Cronbach’s alpha ranging from 0.79 to 0.77) and good internal consistency reliability for the subscale scores (Cronbach’s alpha ranging from 0.93 to 0.74) in three samples. Only the Divergence subscale had a somewhat lower reliability estimate (Cronbach’s alpha ranging from 0.66 to 0.59 across studies). Construct validity was supported, in part, by finding acceptable model-data fit for confirmatory factor analysis (CFA) models in both United States samples and a Greek sample [16-18,21,22]. Acceptable fit indices were found for both a first-order structure (in which the individual items loaded on four factors) and a higher order structure (in which the four first-order factors were indicators of a more global higher order construct). Construct validity was also supported by the association of EBPAS scores with mental health clinic structures and policies [18], culture and climate [23], and leadership [24]. Evidence of content validity was obtained by asking an expert panel to rate the relevance of each item of the EBPAS for each proposed construct [16]. Content validity was supported because every item was on average rated as at least moderately relevant, important, and representative of the factor it was purported to assess. Criterion validity was supported by studies showing that EBPAS scores predict adoption and use of evidence-based interventions [14,25,26]. To date, two studies have examined changes over time of EBPAS scores, with both reporting little variation over time [27,28].

Furthermore, several studies have examined EBPAS scores in relation to individual differences between professionals (e.g. education, level of experience, age, discipline, and sex) and organizational characteristics (e.g. structures and policies, climate and culture, and leadership). Higher educational attainment was associated with a lower likelihood of adopting evidence-based interventions if required, greater willingness to adopt given the appeal of evidence-based interventions, and more general openness to evidence-based interventions [16,24,29,30]. As with educational attainment, years of experience was associated with lower Requirements scores but also associated with lower Openness and EBPAS total scores [16,29]. Years of experience was related to higher Divergence scores for autism early intervention professionals [29] and lower Divergence scores for mental health professionals [16]. Contrasting educational attainment with years of experience demonstrated different patterns suggesting a more restrained openness to adopting an evidence-based intervention for those with higher educational attainment and lower enthusiasm for an evidence-based intervention given more on-the-job experience [16]. Results concerning differences by age, discipline, and sex were inconsistent. Some studies found that older professionals had higher Requirements [16] and Openness scores [23], while other studies found that younger professionals had higher EBPAS total scores [21,31]. However, older professionals also had higher Divergence scores [23]. The relationship between age and attitude toward evidence-based interventions is possibly affected by job tenure. Concerning discipline, one study did not find any difference in EBPAS scores [18], but another study in a United States nationally representative sample found that professionals trained in social work had higher Openness and EBPAS total scores than professionals trained in psychology [16]. Sex differences were absent in two studies [18,21], but in two other studies women had higher Appeal, Requirements, and EBPAS total scores [16,24].

Although the EBPAS has been translated to different languages and is currently being used in several countries (e.g. in Iran, Israel, Japan, Korea, Norway, Romania, and Sweden), a Dutch version of the EBPAS has not yet become available [32]. Further, all research on the psychometric properties of the EBPAS within youth care has been carried out in the United States. It is imperative to test the EBPAS in other countries to facilitate cross-cultural comparisons. Even though there are similarities between youth care in the Netherlands and the United States, such as types of disorders treated, substantial differences exist regarding organizational structures, financial barriers, types of services, training and background of professionals, and client attitudes [33].

The purpose of this study was to investigate the psychometric properties of the Dutch version of the EBPAS. We first translated the EBPAS into Dutch and evaluated the forward and back-translation. Second, we examined the factor structure and internal consistency reliability of the Dutch version of the EBPAS in a diverse sample of youth care professionals. We hereby replicated the exploratory factor analysis and confirmatory factor analyses by Aarons et al. [16] and Aarons [18]. In addition, we conducted a confirmatory bifactor analysis to evaluate the plausibility of subscales, to determine the extent to which the EBPAS total score reflects a single factor, and to evaluate the feasibility of applying a unidimensional model structure to a measure with heterogeneous indicators. Third, we examined the association of EBPAS scores with age, sex, and educational level of the professionals. Based on the literature [32], it was hypothesized that (1) responses to the EBPAS items could be explained by one general factor (attitude toward evidence-based interventions) plus four specific factors (Requirements, Appeal, Openness, and Divergence), (2) good to excellent internal consistency reliability would be found, (3) EBPAS scores would vary by age (with older professionals scoring higher on the Requirements, Openness, and Divergence subscales and lower on the EBPAS total score), (4) EBPAS scores would vary by sex (with women scoring higher on the Appeal and Requirements subscales and EBPAS total score), and (5) EBPAS scores would vary by educational level (with professionals with university education scoring higher on the Appeal and Openness subscales and lower on the Requirements subscale).

## Methods

### Setting

The present study took place within the Academic Center Youth Nijmegen (ACYN), a multidisciplinary collaboration between the Radboud University and multiple youth care institutions in the South-East of the Netherlands. One of the main aims of ACYN is improving the care for youth by making this care more evidence based. Among other things, ACYN stimulates the youth care institutions to use more evidence-based interventions and innovations such as ROM with feedback to clinicians. The coordinators of seven youth care institutions were contacted and the study was described to them in detail. Permission was sought to survey the professionals of the collaborating departments. Five institutions agreed to participate: one large mental healthcare institution and four institutions for child welfare.

### Procedure

With permission of the original author (GAA), a Dutch version of the EBPAS was constructed. First, a forward translation was conducted. The EBPAS was translated into Dutch by the first and last author of this article. The authors emphasized conceptual equivalence of the questionnaire rather than literal translation. Then, a back-translation was made by a bilingual, native English-speaking translator. The first and last author of this article compared the back-translation with the original version and discussed the differences. Subsequently, another bilingual, native English-speaking translator made a literal Dutch translation of the original version, compared the first Dutch translation with the second Dutch translation, and compared the differences with the original version of the EBPAS. It was concluded that no significant differences appeared during the translation process. Only small adjustments were suggested, which were incorporated by the first and last author of this article. The final Dutch version of the EBPAS can be obtained from the first author.

For each participating institution an appointment was made to discuss how and when the EBPAS was assessed. The first two institutions and one department of the third institution preferred a pen and paper survey of the EBPAS. The coordinators of these institutions and this department scheduled the survey sessions within regular team meetings. The surveys were administered to the whole group of professionals at these meetings. After the meetings, the professionals returned the survey in an envelope which was sent to the principal investigator. The other department of the third institution and the fourth and fifth institution preferred a web-based survey of the EBPAS. The professionals of this department and these institutions received a personalized survey invitation email with a web-link to electronic questionnaire system NetQuestionnaires version 6.5 [34]. Professionals who did not respond within a week received a reminder email. After the surveys were completed, the answers were automatically saved in the electronic questionnaire system. The data were exported to an SPSS file. Data collection was conducted from November 2011 through November 2012.

After the data collection was finished, the coordinators of the institutions received an overview of participating professionals. Subsequently, the coordinators collected information about the age, sex, and education of the professionals through the electronic personal files of the institutions. If demographic information was missing, the coordinators directly asked the professionals to provide the information. Due to technical issues with retrieving information from the electronic personal files and in reaching professionals of specific locations, as well as absence of professionals after filling out the EBPAS, it was not possible to obtain demographic information of all professionals.

### Participants

A total of 276 youth care professionals completed the survey. The results of six professionals were excluded; one endorsed two answers for most items, one completed the survey twice, one was not a clinician, and three filled out 0 (Not at all) for all items. Filling out Not at all for every item (straight lining), produced inconsistent results such as “I do not know better than academic researchers how to care for my clients” contrary to “I am not willing to use new and different types of therapy/interventions developed by researchers”. Thus, the final sample size consisted of 270 professionals.

Of the respondents, 140 (51.9%) worked in a mental healthcare institution and 130 (48.1%) worked in a child welfare institution. Data on age, sex, and educational level were missing for 85 (31.5%), 30 (11.1%), and 44 (16.3%) respondents, respectively. The mean age of the remaining respondents was 43.15 years (SD *=* 11.03; range, 23–63) and 71.3% (n = 171) were female. The respondents’ primary discipline was education (n = 78; 34.5%), psychology (n = 66; 29.2%), nursing (n = 33; 14.6%), social work (n = 12; 5.3%), medicine (psychiatrists and physicians; n = 12; 5.3%), non-verbal therapies (e.g. psychomotor therapy; n = 10; 4.4%), teaching (n = 8; 3.5%), and other (n = 7; 3.1%). Because of the relatively few professionals within each discipline, we created groups based on level of education. This resulted in two groups: higher vocational education (education, nursing, social work, non-verbal therapies, and teaching; n = 125; 55.3%) and university education (education, psychology, and medicine; n = 94; 41.6%). The ‘other’ category was excluded from the analyses (n = 7; 3.1%).

### Analyses

SPSS statistical software version 20 [35] was used for the assessment of internal consistency reliability and the exploratory factor analysis. Mplus software version 6 [36] was used for the confirmatory factor analyses, confirmatory bifactor analysis, and multiple linear regression analyses. For one respondent (0.4%), data of seven questions were missing. These missing data were handled in SPSS by imputing the missing values through expectation maximization, which makes use of all available data in estimating parameters. We used the imputed dataset for our analyses. Because we used two different data collection methods, we also tested for differences in mean total score between the pen and paper group and the web-based group in SPSS. Since we found no differences we have merged the results of both groups for the analyses.

We examined descriptive statistics (means and standard deviations) at the item level, for the EBPAS subscales, and the EBPAS total score. We also examined item-total correlations. Internal consistency reliability was examined using Cronbach’s alpha.

To assess the factor structure of the Dutch version of the EBPAS, we first replicated the analyses by Aarons et al. [16] and Aarons [18]. Two separate factor analytic procedures were conducted. The sample was divided by randomly selecting approximately 50% of cases and assigning cases to either an exploratory (n *=* 127) or confirmatory (n *=* 143) analysis group. An exploratory factor analysis (EFA) was conducted on the one half of the sample using Principal Axis Factoring in order to partition systematic and error variance in the solution [37,38]. Promax oblique rotation was used allowing for factor intercorrelations [37]. Items were retained on a factor if they loaded at least 0.32 on the primary factor and less than 0.32 on all other factors [39]. Item-total correlations and scale reliabilities were also used to assess scale structure. Confirmatory factor analyses CFA were conducted on the other half of the sample to test the factor structure derived in the EFA. Because professionals were nested within institutions, models were adjusted for the nested data structure using the TYPE = COMPLEX procedure within MPlus (cf. [40]).

In addition, we conducted a confirmatory bifactor analysis with the following features [41,42]: (1) each item had a non-zero loading on both the general factor and the specific factor that it was designed to measure, but zero loadings on the other specific factors, (2) the specific factors were uncorrelated with each other and with the general factor, and (3) all error terms associated with the items were uncorrelated. The model was identified by fixing the variance of each latent factor to 1. Because the observed variables are measured on a 5-point Likert scale Robust Maximum Likelihood estimation was used.

Commonly accepted rules of thumb for fit indices in confirmatory factor analyses include a comparative fit index (CFI) and Tucker–Lewis Index (TLI) value near 0.95 or greater, a root mean square error of approximation (RMSEA) value near 0.06 or less, and a standardized root mean square residual (SRMR) near 0.08 or less [43].

After identifying a measurement model with acceptable fit, we examined the association of the Dutch EBPAS subscale and total scores with characteristics of the youth care professionals (i.e. age, sex, and educational level). Rather than replicating Aarons [18] multiple stage analytic approach, we replicated Aarons et al. [16] regression analyses because we already adjusted for nested data with the TYPE = COMPLEX procedure within MPlus. We used two-tailed tests.

## Results

### Exploratory factor analysis (EFA)

An EFA was conducted using data from the randomly selected half of the sample (n = 127). The EFA suggested a four-factor solution in accordance with simple structure criteria, scale reliabilities, and parallel analysis. The EFA model accounted for 61% of the variance in the data. Table 1 shows overall means and standard deviations, item-total correlations, eigenvalues, internal consistency reliabilities, and item loadings for each of the scales. Cronbach’s alphas ranged from 0.67 to 0.89, showing acceptable to good values for the different subscales. The overall scale alpha was 0.72. The factors represented four subscales of attitudes toward adoption of evidence-based interventions in keeping with the hypothesized dimensions: Requirements (three items, α = 0.89), Appeal (four items; α = 0.75), Openness (four items; α = 0.77), and Divergence (four items; α = 0.67). Item analyses showed that the reliability coefficient for all the subscales would not significantly improve by removing items from the subscale.

**Table 1:** **EBPAS subscale and item means, standard deviations, item-total correlations, eigenvalues, Cronbach’s alpha, and exploratory factor analysis loadings**

**EBPAS subscales and total**	**M**	**SD**	**Item-total correlation**	**EV**	**α**	**Scale 1**	**Scale 2**	**Scale 3**	**Scale 4**
Requirements	2.38	0.78		4.19	0.89				
Agency required	2.36	0.86	0.67			0.97			
Supervisor required	2.41	0.84	0.60			0.85			
State required	2.37	0.87	0.63			0.76			
Appeal	3.13	0.54		1.94	0.75				
Makes sense	3.22	0.71	0.44				0.84		
Intuitively appealing	3.09	0.78	0.46				0.74		
Get enough training to use	3.18	0.68	0.50				0.37		
Colleagues happy with intervention	3.03	0.71	0.53				0.60		
Openness	2.63	0.62		1.56	0.77				
Will follow a treatment manual	2.74	0.77	0.59					0.66	
Like new therapy types	2.57	0.78	0.43					0.58	
Therapy developed by researchers	2.70	0.78	0.57					0.74	
Therapy different than usual	2.51	0.87	0.60					0.52	
Divergence	1.46	0.65		1.41	0.67				
Research-based treatments not useful	1.15	0.86	0.41						0.48
Will not use manualized therapy	0.81	0.89	0.43						0.44
Clinical experience more important	2.07	0.91	0.25						0.66
Know better than researchers	1.81	0.98	0.32						0.56
EBPAS total	2.67	0.41			0.72				

n = 270 for means, standard deviations, item-total correlations, and Cronbach’s alpha; n = 127 for exploratory factor analysis and eigenvalues.

SD, Standard deviation; EV, Eigenvalue; α, Cronbach’s alpha; factor loadings *<*0.30 are not shown.

### First-order confirmatory factor analysis (CFA)

**Figure 1:**
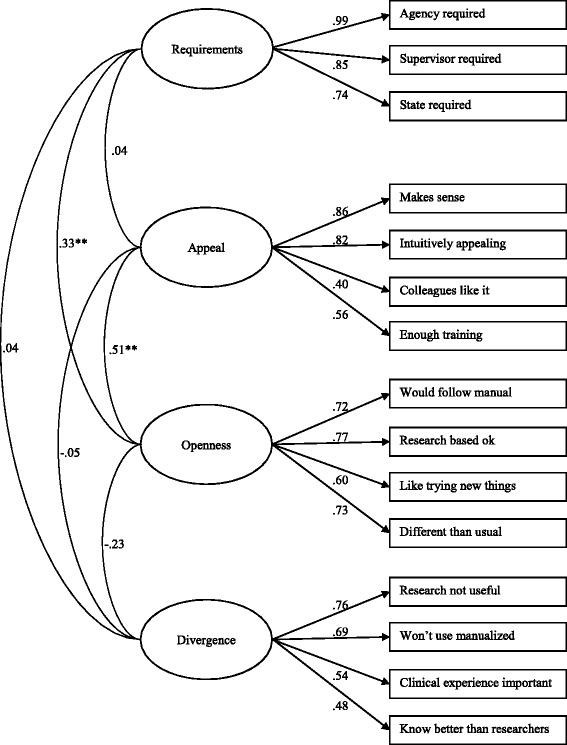
**Confirmatory factor analysis model of the EBPAS.** n = 143, χ^2^ (84) = 179.17, CFI = 0.90, TLI = 0.87, RMSEA = 0.09, SRMR = 0.08, ^*^ *P* < 0.05, ^**^ *P* < 0.01; all factor loadings are significant at *P* < 0.01.

A first-order CFA was conducted using data from the other randomly selected half of the sample (n = 143), specifying the factor structure identified in the EFA. CFA items were constrained to load only on the primary factor indicated in the EFA, thus providing a highly stringent test of the factor structure. As in the EFA, factor intercorrelations were allowed. CFA factor loadings confirmed the EFA-based a priori factor structure and the model demonstrated acceptable fit (χ^2^(84) = 179*.*17, CFI = 0.90, TLI = 0.87, RMSEA = 0.09, SRMR = 0.08) further supporting the original EBPAS factor structure. Factor loadings ranged from 0.40 to 0.99 and all factor loadings were statistically significant. Factor intercorrelations ranged from 0.04 to 0.50. The Openness subscale had a weak positive correlation with the Requirements subscale (*r* = 0.33, *P* <0.01) and a moderate positive correlation with the Appeal subscale (*r* = 0.51, *P* <0.01). The Divergence subscale had no significant correlations with the Requirements (*r* = 0.04, *P* >0.05), Appeal (*r* = −0.05, *P* >0.05), or Openness (*r* = −0.23, *P* >0.05) subscales. The results of the first-order CFA are shown in Figure 1.

### Second-order confirmatory factor analysis (CFA)

**Figure 2:**
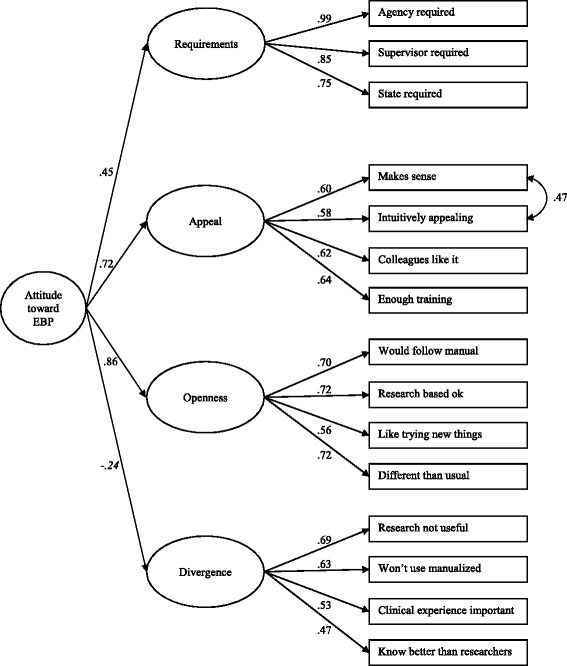
**Second-order confirmatory factor analysis model of the EBPAS.** n = 270, χ^2^ (85) = 146.48, CFI = 0.96, TLI = 0.95, RMSEA = 0.05, SRMR = 0.05; All factor loadings are significant at *P* < 0.01, except for Divergence on Attitude (in italics; *P* = 0.21). Estimation of correlated residuals between two Appeal subscale items is indicated by a double-headed arrow.

In replication of Aarons et al. [16], a second-order CFA was conducted to test if all lower order EBPAS factors are indicators of a higher order construct that might be regarded as general attitude toward adoption of evidence-based interventions. With data from the randomly selected half of the sample that was used for the first-order CFA (n = 143), the model could not be identified. Because we assumed that our subgroup was too small for a second-order CFA, we also conducted the second-order CFA among the total sample of professionals (n = 270). In the total sample, the model could be identified, but the latent variable covariance matrix was not positive definite. This indicated a correlation greater or equal to one between two variables: item 9 (intuitively appealing) and item 10 (makes sense). By correcting the model for this correlation, we found a good fit (χ^2^(85) = 146*.*48, CFI = 0.96, TLI = 0.95, RMSEA = 0.05, SRMR = 0.05) and our results exactly matched the results of Aarons et al. [16]. Factor loadings ranged from 0.45 to 0.99. All factor loadings were statistically significant, except for Divergence on Attitude (*P* = 0.21). The results of the second-order CFA are shown in Figure 2.

### Confirmatory bifactor analysis

**Figure 3:**
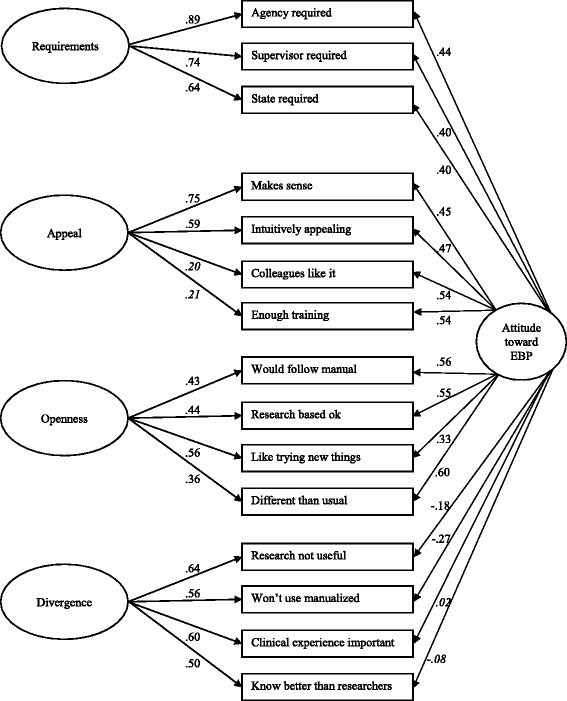
**Confirmatory bifactor analysis model of the EBPAS.** n = 270, χ^2^ (75) = 107.37, CFI = 0.97, TLI = 0.96, RMSEA = 0.04, SRMR = 0.04; All factor loadings are significant at *P* < 0.05, except for items ‘Clinical experience important’ and ‘Know better than researchers’ on the general factor (in italics; *P* = 0.83 and *P* = 0.38), and items ‘Colleagues like it’ and ‘Enough training’ on Appeal (in italics; *P* = 0.16 and *P* = 0.07).

The bifactor model simultaneously assessed the specific factors Requirements, Appeal, Openness, and Divergence as well as the general factor Attitude toward evidence-based interventions shared by those specific factors. The bifactor model demonstrated a good fit (χ^2^(75) = 107.37, CFI = 0.97, TLI = 0.96, RMSEA = 0.04, SRMR = 0.04). The majority of the factor loadings were statistically significant, except for items 3 and 6 on Attitude (*P* = 0.38 and *P* = 0.83), and items 14 and 15 on Appeal (*P* = 0.16 and *P* = 0.07). Most factor loadings (10 out of 15) were weaker for the general factor than for the grouping factors and some loadings were negative. According to Reise et al. 44], if items primarily reflect the general factor and have low loadings on the grouping factors, subscales make little sense. However, when items have substantial loadings on both the general factor and the grouping factors, subscales will make sense. Therefore, the findings indicate that variance is explained both by a general factor (Attitude toward evidence-based interventions) and by specific factors above and beyond the general factor (Requirements, Appeal, Openness, and Divergence). This further supports the original EBPAS factor structure. The results of the confirmatory bifactor analysis are shown in Figure 3.

### Regression analyses

Regression models using the maximum likelihood estimation with robust standard errors were used to assess the association of EBPAS scores with characteristics of youth care professionals. For these analyses, in order to enable a good comparison with the study of Aarons et al. [16], the whole sample was used in a first-order factor model. A second-order factor model could not be identified because the number of iterations was exceeded. The results are shown in Table 2. For the Requirements subscale, willingness to adopt evidence-based interventions given the requirements to do so decreased with higher levels of education. Controlling for age and sex, professionals with university education scored significantly lower than professionals with higher vocational education. For the Appeal subscale, no associations with characteristics of youth care professionals were found. For the Openness subscale, greater openness to new practices was associated with higher levels of education. Controlling for age and sex, professionals with university education scored significantly higher than professionals with higher vocational education. Finally, for the Divergence subscale, greater divergence between evidence-based interventions and current practice was associated with being older, being male, and lower levels of education. While holding constant other variables in the model, older professionals scored significantly higher than younger professionals, males scored significantly higher than females, and professionals with higher vocational education scored significantly higher than professionals with university education.

**Table 2:** **Association of characteristics of youth care professionals with EBPAS scores**

	**Requirements (R** ^**2**^ **= 0.01)**			**Appeal (R** ^**2**^ **= 0.02)**			**Openness (R** ^**2**^ **= 0.06)**			**Divergence (R** ^**2**^ **= 0.15)**		
**Variable**	***ß***	***b***	***z***	***ß***	***b***	***z***	***ß***	***b***	***z***	***ß***	***b***	***z***
Age	0.025	0.001	0.32	–0.031	−0.001	−0.54	–0.153	−0.003	−1.11	0.262	0.005	4.18**
Sex	0.039	0.047	1.18	–0.137	−0.141	−1.70	–0.153	−0.112	−1.62	0.223	0.170	1.97*
Educational level	–0.101	−0.100	−2.99**	0.060	0.051	1.31	0.137	0.082	3.93**	–0.232	−0.145	−3.05**

Reference groups for dummy-coded variables are as follows: sex reference is female and discipline reference is higher vocational education.

*P <0.05, ** *P* <0.01.

## Discussion

The current study contributes to the development and cross-validation of the EBPAS by examining the psychometric properties of the Dutch version of the EBPAS. Besides that, the present research is the first to assess a bifactorial solution of attitude toward evidence-based interventions. The results provide strong support for a structure with a general factor plus four specific factors of the Dutch version of the EBPAS in a diverse sample of youth care professionals of both a mental healthcare institution and institutions for child welfare. The general factor is attitude toward evidence-based interventions and the specific factors involve willingness to adopt evidence-based interventions given the intuitive appeal, willingness to adopt evidence-based interventions if required, general openness toward evidence-based interventions, and perceived divergence of usual practice with evidence-based interventions. The EBPAS total score and subscale scores demonstrated acceptable to good internal consistency reliability and the mean loading of items on their predicted factors was substantial to good. These findings are highly congruent with studies of the original version of the EBPAS conducted in the United States [32].

Generally consistent with previous findings, Openness subscale scores were positively correlated with Appeal and Requirements subscale scores. Professionals who are more open toward adoption of evidence-based interventions may also endorse positive attitudes toward the intuitive appeal of these interventions and the requirements to adopt these interventions. Contrary to previous findings, we did not find a statistically significant correlation between the extent to which professionals would adopt evidence-based interventions if they were intuitively appealing and if they were required. The Divergence subscale was not significantly correlated with any of the other subscales. Therefore, the extent to which professionals perceive evidence-based interventions as not clinically useful does not appear to be meaningfully associated with openness to evidence-based interventions, appeal of these interventions, and willingness to adopt these interventions if required. Moreover, we did not find a statistically significant correlation between the Divergence subscale and the general Attitude toward adoption of evidence-based interventions. While the three distinct dimensions Requirements, Appeal and Openness possibly address a common attitude toward evidence-based interventions, Divergence seems to be a separate construct in the Dutch sample.

Possible explanations for the finding that the Divergence factor loading was not statistically significant in the higher order model might lie in cultural, political and/or time differences between youth care institutions in the United States and the Netherlands. A possible cultural difference is that Dutch professionals may view research as separate from using evidence-based interventions [45,46]. There are three items in the EBPAS that explicitly use the term “research/researchers”: two items of the Divergence subscale (“Research-based treatments/interventions are not clinically useful” and “I know better than academic researchers how to care for my clients”) and one item of the Openness subscale (“I am willing to use new and different types of therapy/interventions developed by researchers”). The item scores of the Dutch sample appear to be somewhat higher on the first two items and somewhat lower on the last item compared to scores found in the United States samples. Dutch professionals may feel more discrepancy between their clinical views and scientific results or perceive research as confining their ability to make independent decisions about therapy, while they still can be open to evidence-based interventions, can find these interventions appealing, and are willing to adopt these interventions if required. A possible time difference is that evidence-based interventions may be more widely disseminated and implemented in the United States than in the Netherlands. The main body of research about attitudes toward evidence-based interventions (beginning in 2004) is from the United States, exposure to the concept and meaning of evidence-based interventions may be more common across different professional disciplines (e.g. social work, psychology, marriage and family therapy, nursing) in the United States, and this has been developing for a longer period of time than in the Netherlands.

The results also demonstrated associations between EBPAS scores and characteristics of youth care professionals. Professionals with university education scored higher on Openness and lower on both Requirements and Divergence than professionals with higher vocational education. Professionals with university education may have received more education related to evidence-based interventions. In addition, it is likely that the drawbacks of overreliance upon unsystematic clinical observations and experience alone, were raised. It may also be that professionals with university education have had more training and applied practice in evidence-based interventions. Professionals whose education includes and emphasizes exposure to and training in research may be more likely to understand, value, and utilize research [47]. Such professionals may report more openness and less divergence. However, professionals with university education also seem less willing to adopt evidence-based interventions if required. It may be that professionals with additional education and practice are more autonomous and assertive in making independent decisions about utilizing evidence-based interventions [16] and therefore less willing to accept external pressure than their colleagues with lower education.

Besides this, males and older professionals reported more perceived divergence than females and younger professionals. The sex difference is in contrast to studies with the original version of the EBPAS, in which this effect was not found [32]. This suggests that additional research is needed to explore when, where, and why sex differences in attitudes toward evidence-based interventions might operate. The age difference is in accordance with one earlier study [23]. It is conceivable that younger professionals, like professionals with a university degree, are more familiar with evidence-based interventions because they have had more prior exposure to these interventions during their education than older professionals [48]. Being older may result in giving more importance to own clinical experience and less appreciation of research-based interventions.

Some limitations should be noted. First, the sample was not large and thus statistical power was limited. Further, data on age was missing for almost one-third of the respondents and data about level of experience was not available, limiting the direct comparisons that could be made with previous studies. Additionally, only one mental healthcare organization was included; although this was a large one, caution should be exercised when interpreting the generalizability to other mental healthcare institutions. Furthermore, while our sample consisted of professionals of a mental healthcare institution and institutions for child welfare, our findings may not generalize to professionals in individual practice or to other sectors. Finally, because only information about respondents was available, it was not possible to compare respondents with non-respondents to examine a potential non-response bias.

This study has added to the knowledge about the EBPAS in general and for the Netherlands specifically. To our knowledge, this is the first study that used the EBPAS in the Netherlands. Additional research is needed to further establish the factor structure of the Dutch version of the EBPAS. Both the first-order structure and higher order structure require more consideration and investigation. Additionally, it is not clear if the factor structure would vary for child mental health versus child welfare professionals. Future research should examine factor structure differences by professional primary discipline, level of experience, and sector. In addition, the construct and criterion validity of the Dutch version of the EBPAS have to be further established. More research is needed to confirm associations between EBPAS scores and characteristics of youth care professionals, structures and policies of youth care institutions, culture and climate, and leadership. Furthermore, the sensitivity to change of the Dutch version of the EBPAS should be investigated to be able to examine how attitudes towards evidence-based interventions might influence behaviour, how behaviour might influence change in attitudes, and whether attitudes predict adoption, implementation, and sustainment of evidence-based interventions. Finally, for implementation of evidence-based interventions within youth care, it will be useful to examine factors that might moderate the relationship between attitudes and behaviour. Such behaviours are not limited to the use of efficacious treatment models, but can also include use of innovations such as data monitoring systems, alerts to target prescribing practices, and ROM. Although the referent for the EBPAS are interventions that have research support and may be manualized and structured, attitudes towards these interventions can also reflect attitudes toward other clinical innovations. Using information obtained by the Dutch version of the EBPAS may ultimately enable a better tailoring of implementation efforts to the readiness of youth care professionals to adopt evidence-based initiatives.

## Conclusions

The present study provides strong support for the original four-factor structure and internal consistency reliability of the Dutch version of the EBPAS in a diverse sample of youth care professionals. This supports the utility of the EBPAS in varied countries and settings. Because we focused on real-world professionals, the study also provides support for ecological validity.

The study suggests four directions for future research. First, the factor structure of the Dutch version of the EBPAS needs to be further established. Second, additional research is needed to further establish the construct and criterion validity of the Dutch version of the EBPAS. Third, the sensitivity to change of the Dutch version of the EBPAS should be investigated. Finally, it is recommended to examine factors that might moderate the relationship between attitudes and behaviour of youth care professionals.

## Abbreviations

ACYN: Academic Center Youth Nijmegen
CFA: Confirmatory factor analysis
CFI: Comparative fit index
EBP: Evidence-based practice
EBPAS: Evidence-Based Practice Attitude Scale
EFA: Exploratory factor analysis
RMSEA: Root mean square error of approximation
ROM: Routine Outcome Monitoring
SRMR: Standardized root mean square residual
TLI: Tucker–Lewis Index
